# Statin use in relation to long-term survival after gastrectomy for gastric adenocarcinoma: a Swedish population-based cohort study

**DOI:** 10.1007/s10120-024-01487-5

**Published:** 2024-03-02

**Authors:** Dag Holmberg, Joonas H. Kauppila, Johannes Asplund, Wilhelm Leijonmarck, Fredrik Mattsson, Jesper Lagergren

**Affiliations:** 1 Department of Molecular Medicine and Surgery, Karolinska Institutet and Karolinska University Hospital, Retzius Street 13A, 4th Floor, 171 77 Stockholm, Sweden; 2 Department of Surgery, Oulu University Hospital and University of Oulu, Oulu, Finland; 3 School of Cancer and Pharmacological Sciences, King’s College London, London, UK

**Keywords:** Chemoprevention, Gastric neoplasm, Gastrectomy, Hyperlipidaemia

## Abstract

**Background:**

Studies have suggested that medication with statins improves survival in patients with gastric cancer, but methodological issues have limited the interpretability and prohibited conclusive results. We aimed to provide valid evidence as to whether statin use improves survival of gastric adenocarcinoma.

**Methods:**

This nationwide and population-based cohort study included virtually all patients who underwent curatively intended surgery (gastrectomy) for gastric adenocarcinoma in Sweden between 2006 and 2015 with follow-up throughout 2019 for disease-specific mortality and 2020 for all-cause mortality. Data came from medical records and national healthcare registries. The exposure was statin use during the year prior to gastrectomy which was compared to no such use during the same period. The outcomes were 5-year disease-specific mortality (main) and 5-year all-cause mortality (secondary). Multivariable Cox regression provided hazard ratios (HR) with 95% confidence intervals (CI), adjusted for age, sex, education, calendar year, comorbidity, low-dose aspirin use, tumour sublocation, pathological tumour stage, neoadjuvant chemotherapy, annual surgeon volume, and surgical radicality.

**Results:**

Among 1515 participating patients, the mean age was 69 years and 58.4% were men. Statin use, identified in 399 (26.3%) patients, was not associated with any statistically significantly decreased 5-year disease-specific mortality **(**HR 0.99, 95% CI 0.82–1.21) or 5-year all-cause mortality (HR 0.94, 95% CI 0.79–1.12). No risk reductions were found across subgroups of age, sex, aspirin user status, or tumour stage, or in patients with long-term preoperative of postoperative use of statins, all with point estimates close to 1.

**Conclusions:**

Perioperative use of statins does not seem to improve the 5-year survival in patients who undergo gastrectomy with curative intent for gastric adenocarcinoma in Sweden.

**Supplementary Information:**

The online version contains supplementary material available at 10.1007/s10120-024-01487-5.

## Introduction

Gastric cancer (> 95% adenocarcinoma) is the 3rd most common cause of cancer deaths globally [1]. Gastric adenocarcinoma often presents at an incurable stage in Sweden, where there is no population screening, rendering an overall 25% 5-year survival rate [2, 3]. Surgery with total or subtotal gastrectomy (often combined with chemotherapy) is the main curative treatment for gastric adenocarcinoma in Sweden. The 5-year survival in patients who undergo curatively intended treatment is 40–50% [4, 5].

Statins are medications that decrease serum cholesterol levels to prevent cardiovascular disease through the inhibition of 3-hydroxy-methylglutaryl CoA reductase (HMGCR), the key enzyme in the biosynthesis of cholesterol. Several studies have demonstrated that statins also have anti-carcinogenic properties, where the reduction of cholesterol and inhibition of the mevalonate pathway could counteract cancer cell proliferation and migration, metastases, and cancer-related mortality [6, 7]. Specifically, HMGCR has been found to be upregulated in gastric cancer cell lines, where statins can inhibit proliferation and migration, and promote apoptosis [8]. Similarly, statins prevent cancer development and growth in animal models in various tumour forms [9]. Observational studies in humans have also indicated that statin use is associated with a reduced risk of developing several gastrointestinal cancers, including gastric cancer [7, 10], and possibly also with improved survival once gastric cancer has developed [11–13]. In a recent systematic review and meta-analysis of 6 studies and 5693 patients with gastric cancer, the all-cause mortality was 28% decreased (HR 0.72, 95% CI 0.53–0.97) among statin users, but heterogeneity was substantial (*I*^*2*=^88.0%) [13]. The individual studies have been hampered by methodological issues such as immortal time bias (due to the use of time-dependent definitions of the exposure), confounding by strong prognostic factors that were not accounted for, and short and incomplete follow-up.

Using a population-based cohort design with high participation, comprehensive and high-quality data, and long and complete follow-up, we aimed to provide the best available evidence to date to determine whether statin use improves survival in patients who undergo curatively intended treatment for gastric adenocarcinoma in Sweden.

## Methods

### Design

This was a nationwide Swedish population-based cohort study during the study period 2006 throughout 2020 examining statin use in relation to mortality in patients who underwent curatively intended treatment for gastric adenocarcinoma. Data came from the Swedish Gastric Cancer Surgery Study (SWEGASS), which have been presented in detail in a recent cohort description [14]. In brief, SWEGASS included > 98% of all patients having undergone gastrectomy for gastric adenocarcinoma (including Siewert type III tumours of the gastric cardia) in Sweden from July 1, 2006, to December 31, 2015. Data on the exposure (statin use), were retrieved from the Swedish Prescribed Drug Registry, which electronically and automatically records all prescribed and dispensed drugs in Sweden, except for in-hospital use. The registration started in July 1, 2005 and inclusion started exactly 1 year later to allow assessment of dispensation of statins before surgery. The registry covers all Swedish pharmacies, and the recording is nearly 100% complete [15]. For the present study, the follow-up was updated until December 31, 2020. We excluded other (rarer) histological types of gastric malignancies than adenocarcinoma because of major differences in treatment and survival. Potentially eligible patients were initially identified in the Swedish Cancer Registry and Swedish Patient Registry [16]. The completeness of the registration in the Cancer Registry is 98% for gastric adenocarcinoma overall, and likely even higher for those who undergo gastrectomy [17]. Among all identified patients, the final cohort was selected after a review of medical records, including notes from multidisciplinary meetings, surgery charts, histopathology reports, and hospital discharge summaries [14]. Patients operated without a curative intent were excluded because statins are unlikely to influence survival at non-operable tumour stages. The study was approved by the Regional Ethical Review Board in Stockholm, Sweden (2017/141-31/2).

### Exposure

The study exposure was dispensation of a statin prescribed by a physician within 1 year prior to gastrectomy (or longer), defined by the Anatomical Therapeutic Chemical codes C10AA or C10BA. In separate sensitivity analyses, we examined statin use for 2 years and 3 years preoperatively, as well as statin use during the first postoperative year. All dispensed medications were considered to be taken and dispensation is hereafter termed as “use”. Statins are only available by prescription in Sweden and not over-the-counter.

### Outcomes

The main outcome was 5-year disease-specific mortality, defined as death with gastric cancer as an underlying or contributing cause of death, occurring between the date of gastrectomy and 5 years postoperatively. The secondary outcome was 5-year all-cause mortality, defined as death from any cause within 5 years of the gastrectomy. Information on the mortality outcomes was obtained from the Swedish Cause of Death Registry. This registry has 100% completeness for date of death and 96% completeness for cause of death for all Swedish residents, including deaths among Swedish residents who die abroad [18]. Information on date of death is updated continuously, while causes of death are assembled at the end of each calendar year. Therefore, follow-up for disease-specific mortality ended December 31, 2019, which did not allow 5-year follow-up of all patients, while follow-up for all-cause mortality ended December 31, 2020, allowing 5-year follow-up of all participants.

### Confounders

Eleven covariates (categorisations in brackets) were considered potential confounders: age (continuous, linear modelling), sex (male or female), education (≤ 9, 10–12, or > 12 years of formal education, which represents primary school, secondary school, and tertiary education/university), calendar year (continuous, linear modelling), comorbidity (Charlson comorbidity index score 0, 1, or ≥ 2), low-dose (75–160 mg/day) aspirin use (yes or no), tumour sublocation (cardia or non-cardia), pathological tumour stage (0–I, II, III, or IV), neoadjuvant chemotherapy (yes or no), annual surgeon volume of gastrectomy (quartiles, i.e., four equal-sized groups), and radicality of the surgical resection (R0 or R1, [patients with R2 were excluded]). Information on tumour location, tumour stage, neoadjuvant chemotherapy, surgeon volume, and surgical radicality was retrieved from a review of medical records. Data on age, sex, education, calendar year, comorbidity, and aspirin use were obtained from three nationwide complete Swedish registries: Patient Registry, Longitudinal Integrated Database for Health Insurance and Labour Market Studies (LISA), and Prescribed Drug Registry [15, 16, 19]. Comorbidity was classified based on the most well-validated version of the Charlson comorbidity index (Supplementary Table 1) [20]. Aspirin use was included as a covariate because it is often used alongside statins and may improve gastric cancer survival [21]. Type of surgery (total versus subtotal gastrectomy) was not adjusted for due to collinearity with tumour sublocation, which was included in the main model. Postoperative complications were presented but not adjusted for, because these occurred after baseline (date of surgery).

### Statistical analysis

Study patients were followed from the date of gastrectomy until death, 5 years after surgery, or end of the study period, whichever occurred first. The cumulative survival as a function of time was estimated using the Kaplan–Meier estimator for a descriptive comparison of users and non-users of statins (Supplementary Figs. 1 and 2). Cox proportional hazards models were used to calculate hazard ratios (HR) with 95% confidence intervals (CI) of the mortality outcomes among statin users compared to non-users of statins (reference group in all analyses). A multivariable model adjusted for the 11 covariates, described and categorised above. Effect modification analyses were conducted for covariates where a differential effect of statins was plausible. This analysis was conducted by including an interaction term in the models for the subgroups of age (≤ 66, 67–74, and ≥ 75 years), sex (male or female), low-dose aspirin use (yes or no), and pathological tumour stage (0–I, II, III, and IV), for which HRs with 95% CI were presented. For the interaction analyses, the variables were categorised. A likelihood-ratio test for each interaction term at the 0.05 level was computed by calculating the difference between the log likelihood statistics in the main adjusted model and the main adjusted model including the interaction term. To manage missing data, we used complete case analysis, i.e., excluded patients with any missing data in the models. Missing data were found in at least one of the 11 covariates in 10% of patients (*n* = 167) (education, *n* = 35; surgeon volume, *n* = 7; pathological tumour stage, *n* = 36; neoadjuvant chemotherapy, *n* = 6; tumour sublocation, *n* = 7; surgical radicality, *n* = 95). The proportional hazards assumption was evaluated using log–log survival plots and by calculating the correlations between Schoenfeld residuals for the covariates and ranking of individual failure time. The correlations were low, indicating that the proportional hazards assumption was met for all analyses. A senior biostatistician (FM) conducted the data management and statistical analyses according to a detailed and pre-defined study protocol and used the components SAS/BASE and SAS/STAT in the SAS software, Version 9.4 (SAS Institute Inc., Cary, NC, USA) for these purposes.

## Results

### Patients

**Fig. 1:**
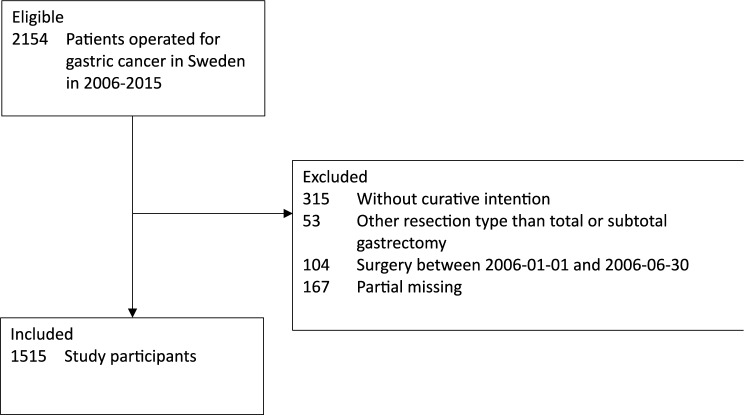
Selection of study participants

Figure 1 is a flowchart of how the 1515 final study patients were included in the study and Table 1 shows these patients’ characteristics. The mean age at the time of surgery was 69 years, and 58.4% (*n* = 885) were men. Compared to non-users of statins, statin users were slightly older, more frequently men, had more comorbidities, and were more often users of low-dose aspirin. Tumour characteristics, such as tumour stage and subsite, as well as surgery characteristics, such as surgeon volume and surgical radicality, were similar between users and non-users of statins.

**Table 1:** Characteristics of 1515 patients who underwent curatively intended gastrectomy for gastric adenocarcinoma

	**Number (%)**	
	**Users of statins**	**Non-users of statins**
**All patients**	399 (100.0)	1116 (100.0)
**Age (years)**		
Mean (standard deviation)	73 (8)	68 (12)
< 65	63 (15.8)	434 (38.9)
66–74	148 (37.1)	323 (28.9)
≥ 75	188 (47.1)	359 (32.2)
**Sex**		
Men	255 (63.9)	630 (56.5)
Women	144 (36.1)	486 (43.5)
**Education (years)**		
< 9	192 (48.1)	437 (39.2)
10–12	163 (40.9)	458 (41.0)
> 12	44 (11.0)	221 (19.8)
**Calendar period of surgery (years)**		
2006–2010	189 (47.4)	547 (49.0)
2011–2015	210 (52.6)	569 (51.0)
**Charlson comorbidity index score**		
0	94 (23.6)	581 (52.1)
1	138 (34.6)	353 (31.6)
≥ 2	167 (41.8)	182 (16.3)
**Low-dose aspirin use**		
Yes	243 (60.9)	156 (14.0)
No	156 (39.1)	960 (86.0)
**Tumour sublocation**		
Cardia (Siewert type III)	43 (10.8)	114 (10.2)
Non-cardia	356 (88.2)	1002 (89.8)
**Pathological tumour stage**		
0-I	117 (29.3)	313 (28.1)
II	139 (34.8)	339 (30.4)
III	131 (32.8)	413 (37.0)
IV	12 (3.0)	51 (4.5)
**Neoadjuvant chemotherapy**		
Yes	93 (23.3)	415 (37.2)
No	306 (76.7)	701 (62.8)
**Type of surgery**		
Total gastrectomy	170 (42.6)	552 (49.5)
Subtotal gastrectomy	229 (57.4)	564 (50.5)
**Mean annual surgeon volume of gastrectomy**		
< 5	266 (66.7)	714 (64.0)
5–9	109 (27.3)	328 (29.4)
≥ 10	24 (6.0)	74 (6.6)
**Surgical radicality**		
Radical (R0)	366 (91.7)	1006 (90.1)
Not microscopically radical (R1)	33 (8.3)	110 (9.9)
**Postoperative complications (Clavien–Dindo)**		
No	211 (52.8)	705 (63.2)
I	88 (22.1)	227 (20.3)
II	59 (14.8)	122 (10.9)
≥ III	41 (10.3)	62 (5.6)

### Statin use and risk of 5-year mortality

The cumulative incidence of 5-year all-cause mortality was 59.4% (*n* = 237) among statin users and 57.9% (*n* = 646) among non-users of statins. The majority of patients died with gastric cancer as an underlying or contributing cause of death (*n* = 197; 83.1% of statin users and *n* = 563; 87.2% in non-users). The multivariable regression analyses showed no decreased risk of 5-year disease-specific mortality among statin users (adjusted HR 0.99, 95% CI 0.82–1.21) (Table 2). The overall adjusted HR of for 5-year all-cause mortality was 0.94 (95% CI 0.79–1.12). The lack of decreased risk was consistent across age groups and sexes, among low-dose aspirin users and non-users, as well as tumour stages, with point estimates close to 1 and without evidence of heterogeneity (Table 2 and Supplementary Table 2). The sensitivity analyses did not reveal decreased 5-year disease-specific mortality in patients using statins for 2 years preoperatively (adjusted HR 0.96, 95% CI 0.78–1.19) or 3 years preoperatively (adjusted HR 0.86, 95% CI 0.67–1.11), nor in patients using statins postoperatively (adjusted HR 1.06, 95% CI 0.81–1.39).

**Table 2:** Statin use and hazard ratio (HR) with 95% confidence interval (CI) of mortality among 1515 patients who underwent gastrectomy with curative intent for gastric adenocarcinoma

				**5-year disease-specific mortality**		**5-year all-cause mortality**	
	**At risk (n)**	**Person-years**	**Deaths (n)**	**Unadjusted HR (95% CI)**	**Adjusted HR (95% CI)**^**a,b**^	**Unadjusted HR (95% CI)**	**Adjusted HR (95% CI)**^**a,b**^
**Total cohort**							
No statin use	1116	3446	646	1.00 (reference)	1.00 (reference)	1.00 (reference)	1.00 (reference)
Statin use	399	1203	237	1.00 (0.85–1.18)	0.99 (0.82–1.21)	1.05 (0.91–1.22)	0.94 (0.79–1.12)
** ≤ 65 years**							
No statin use	434	1463	218	1.00 (reference)	1.00 (reference)	1.00 (reference)	1.00 (reference)
Statin use	63	230	30	0.88 (0.59–1.31)	0.88 (0.58–1.33)	0.88 (0.60–1.29)	0.81 (0.55–1.21)
**66–74 years**							
No statin use	323	977	192	1.00 (reference)	1.00 (reference)	1.00 (reference)	1.00 (reference)
Statin use	148	452	82	0.97 (0.74–1.27)	1.10 (0.83–1.47)	0.94 (0.72–1.21)	0.98 (0.75–1.29)
** ≥ 75 years**							
No statin use	359	1006	236	1.00 (reference)	1.00 (reference)	1.00 (reference)	1.00 (reference)
Statin use	188	520	125	0.95 (0.74–1.22)	0.95 (0.73–1.24)	1.02 (0.82–1.27)	0.95 (0.75–1.20)
**Men**							
No statin use	630	1932	364	1.00 (reference)	1.00 (reference)	1.00 (reference)	1.00 (reference)
Statin use	255	752	158	1.03 (0.84–1.27)	0.94 (0.74–1.19)	1.11 (0.92–1.33)	0.92 (0.75–1.14)
**Women**							
No statin use	486	1514	282	1.00 (reference)	1.00 (reference)	1.00 (reference)	1.00 (reference)
Statin use	144	450	79	0.95 (0.73–1.25)	1.09 (0.82–1.44)	0.96 (0.75–1.23)	0.96 (0.84–1.25)
**Low-dose aspirin**							
No statin use	156	463	97	1.00 (reference)	1.00 (reference)	1.00 (reference)	1.00 (reference)
Statin use	243	743	146	1.04 (0.78–1.40)	0.95 (0.71–1.28)	0.94 (0.73–1.21)	0.86 (0.66–1.12)
**No low-dose aspirin**							
No statin use	960	2984	549	1.00 (reference)	1.00 (reference)	1.00 (reference)	1.00 (reference)
Statin use	156	460	91	1.01 (0.79–1.28)	1.02 (0.80–1.31)	1.08 (0.86–1.35)	1.00 (0.80–1.26)
**Tumour stage 0–I**							
No statin use	313	1322	79	1.00 (reference)	1.00 (reference)	1.00 (reference)	1.00 (reference)
Statin use	117	483	36	1.27 (0.75–2.15)	1.20 (0.70–2.05)	1.25 (0.84–1.85)	1.10 (0.74–1.65)
**Tumour stage II**							
No statin use	339	1169	187	1.00 (reference)	1.00 (reference)	1.00 (reference)	1.00 (reference)
Statin use	139	440	81	1.12 (0.84–1.49)	1.04 (0.77–1.41)	1.15 (0.88–1.49)	0.97 (0.73–1.28)
**Tumour stage III**							
No statin use	413	872	334	1.00 (reference)	1.00 (reference)	1.00 (reference)	1.00 (reference)
Statin use	131	262	110	1.05 (0.84–1.32)	0.97 (0.76–1.25)	1.10 (0.89–1.36)	0.92 (0.72–1.17)
**Tumour stage IV**							
No statin use	51	83	46	1.00 (reference)	1.00 (reference)	1.00 (reference)	1.00 (reference)
Statin use	12	18	10	0.98 (0.48–2.00)	0.71 (0.34–1.49)	1.00 (0.51–1.99)	0.60 (0.30–1.22)

^a^Adjusted for age, sex, education level, calendar period, comorbidity, aspirin use, pathological tumour stage, tumour location, neoadjuvant chemotherapy, surgeon volume, and surgical radicality

^b^Age was considered a categorical variable in the interaction analyses

## Discussion

The results from this study indicate that the use of statins for up to 3 years prior and 1 year after curatively intended gastrectomy for gastric adenocarcinoma does not improve the 5-year disease-specific or 5-year all-cause survival in a Swedish setting.

Previous studies have produced inconsistent results regarding the association between statin use and survival in patients with gastric cancer. In a Korean randomised controlled trial of 244 patients with advanced gastric cancer treated with chemotherapy, patients who were randomised to receive an addition of the statin simvastatin 40 mg once daily did not have any longer progression-free survival (HR 0.93, 95% CI 0.68–1.26) or overall survival (HR 0.97, 95% CI 0.72–1.29) than patients receiving placebo, without any apparent benefit in subgroups [22]. A national Korean cohort study of 11,568 patients with endoscopically resected early-stage gastric cancer found that statins strongly reduced the risk of metachronous recurrence (HR 0.17, 95% CI 0.13–0.24) [12]. However, such a powerful effect is at face value improbable, superseding that of virtually any standard oncological treatment and clearly at odds with the results of other studies. The findings may have been influenced by biases related to time at risk and reverse causation, because the study used a time-dependent definition of the exposure. Methodological papers have instead suggested an approach in observational pharmacoepidemiology using a “target trial approach”, e.g., an approach where the observational design is emulating the randomised clinical trial design [23, 24]. This includes using a defined start of follow-up for both exposed and unexposed patients and avoidance of time-dependent analysis to avoid immortal time at risk and reverse causation. Previous studies have often failed in acknowledging these methodological principles and the results of these studies are therefore difficult to interpret. A Spanish prospective non-randomised trial of 60 patients with stage III–IV gastric cancer, of whom 20 were assigned 40 mg pravastatin (statin) once daily in addition to chemotherapy and surgery, without finding any benefit in median overall survival (15 months in the control group vs 14 months in the statin group, *p* = 0.8) [25]. Whereas control of confounding and definitions of the exposure seemed adequate, the study was underpowered to assess a potential difference between the experimental and comparison arm. A population-based cohort study from Taiwan included 367 statin users and 1468 matched non-users of statins with gastric cancer treated with chemotherapy and surgery [26]. The study found a strong and dose-dependent improvement in survival of statin users, but defined statin use based on cumulative prescriptions during the follow-up time, thereby introducing immortal time for statin users. Additionally, tumour stage was not adjusted for, leaving the results susceptible to confounding. A Korean case–control study of 65 statin users and 176 non-users of statin who all underwent gastrectomy for gastric cancer found that short-term (< 6 months) statin use was associated with an increased odds of gastric cancer recurrence, whereas use > 6 months was associated with decreased odds compared to no use. The statin use could be attained any time during the follow-up, thus introducing immortal time [27]. A large population-based cohort study from the United Kingdom of patients with gastric cancer found statin use to be associated with a decreased risk of cancer-specific mortality (HR 0.83, 95% CI 0.74–0.92) [11]. However, that study included patients with non-operable tumours and all histological type of gastric malignancy, and tumour stage was missing in > 80% of patients, rendering the results susceptible to biases [11]. The results of all the aforementioned studies (except for the study on endoscopically resected cancer) were pooled in a recent meta-analysis, showing a statistically significant 28% decreased risk of all-cause mortality in patients with gastric cancer [13]. However, there was significant heterogeneity between the 5 included observational studies (5693 patients) and the included studies contained the methodological issues outlined above.

The findings from the present study do not support the hypothesis that the use of statins improves disease-specific or all-cause survival in patients with curable gastric adenocarcinoma. This study could avoid or at least reduce the major methodological flaws often seen in pharmacoepidemiologic studies, particularly immortal time bias and confounding [24]. The findings were well in line with the only randomised controlled trial on the topic [22], although the present study focused on curatively treated patients.

Methodological strengths include the nationwide and population-based cohort design, which included virtually all patients in Sweden with curatively intended gastrectomy for gastric adenocarcinoma during the study period. We excluded non-adenocarcinoma histological types to avoid bias. Data on the exposure, covariates and outcomes were of high quality and completeness without any losses of follow-up. Results were adjusted for all known prognostic factors in gastric adenocarcinoma. Immortal time bias was avoided by starting follow-up at the date of surgery and disregarding postoperative use of statins, which is in accordance with the intention-to-treat principle, i.e., patients remained in the exposed or unexposed group as classified at baseline throughout the follow-up. The robustness of the results was confirmed by subgroup analyses. There are also weaknesses. Residual confounding by unknown variables cannot be excluded. However, the extensive adjustment for prognostic factors should counteract confounding. We could not confirm that patients actually used the statin medication. However, compliance should be high for these drugs because patients had to have a prescription of a statin from a physician, go to a pharmacy and dispense the statins to be counted as users. In-hospital use of statins could not be measured because such use is not registered in the Prescribed Drug Registry, but it is unlikely that any such use was limited to the in-hospital stay and was unlikely to affect the results. Although the sample size and statistical precision of the main analyses were sufficient, the precision was lower in the subgroup analyses and we cannot exclude an influence of statins in some subgroups. Particularly, the point estimates of 5-year disease-specific were lower in younger individuals and in patients with metastasized disease, but the confidence intervals were wide. The point estimates of the other subgroup analyses were close to 1, which supports the findings from the main analysis. A minority of patients (*n* = 139, 9.2%) were operated in 2015 and thus contributed only between four and five of follow-up, but any influence of this slightly shorter follow-up on the overall results should be negligible. Generalisability of the results to high-risk areas such as East Asia may be limited, because of different healthcare structure, screening practices, demographics and risk factor prevalence. However, the findings should be generalisable to other populations with similar demographic characteristics, healthcare and prevalence of Helicobacter pylori infection as in Sweden, i.e., the Nordic countries.

In conclusion, this nationwide and population-based cohort study with adjustment for confounding, complete and long-term follow-up, and avoidance of immortal time bias found no support for the hypothesis that statin use improves disease-specific or all-cause survival in patients who undergo curatively intended treatment for gastric adenocarcinoma. Thus, statins might not be an effective addition to the treatment of this tumour in Sweden.

### Supplementary Information

Below is the link to the electronic supplementary material.

Supplementary file1 (DOCX 51 KB)
