# Supplementary material for: Statin use in relation to long-term survival after gastrectomy for gastric adenocarcinoma: a Swedish population-based cohort study
Source: Gastric Cancer. 2024 Mar 2;27(3):590–7. doi: 10.1007/s10120-024-01487-5 (PMC11016510; doi:10.1007/s10120-024-01487-5)

| **Supplementary Table 1. Diagnoses and corresponding ICD-codes for Charlson comorbidity index in Sweden.** | |
| --- | --- |
|  | **ICD-10** |
| **Years of use** | 1997-present |
|  |  |
| **Myocardial Infarction** | I21-I23, I252 |
| **Congestive Heart Failure** | I11, I13, I255, I42-I43, I50, I517 |
| **Peripheral Vascular Disease** | I70–I73, I770–I771, K551, K558– K559, R02, Z958–Z959 |
| **Cerebrovascular Disease** | G45–G46, I60–I69 |
| **Dementia** | A810, F00–F03, F051, G30–G31 |
| **Chronic Pulmonary Disease** | I26–I27, J40–J47, J60–J67, J684, J701, J703 |
| **Rheumatic Disease** | M05–M06, M09, M120, M315, M32–M36 |
| **Liver Disease** | B18, I85, I864, I982, K70–K71, K721, K729, K76, R162, Z944 |
| **Hemiplegia** | G114, G81–G83 |
| **Renal Disease** | I12–I13, N01, N03, N05, N07–N08, N171–N172, N18, N19, N25, Z49, Z940, Z992 |
| **Any Malignancy** | C00–C26, C30–C34, C37–C41, C43, C45–C58, C60–C76, C80–C85, C88, C90–C97 |
| **Metastatic Tumors** | C77–C79 |
| **AIDS** | B20–B24 |

**Supplementary Table 2. Results of the heterogeneity test using a likelihood-ratio test for each interaction term.**

**Interaction term P-value**

**Statin*Age** 0.59244

**Statin*Sex** 0.41028

**Statin*Low-dose aspirin**  0.71028

**Statin*Tumour stage** 0.68828

**Supplementary Figure 1. Cumulative incidence of 5-year gastric cancer-specific mortality in patients with and without statins.**


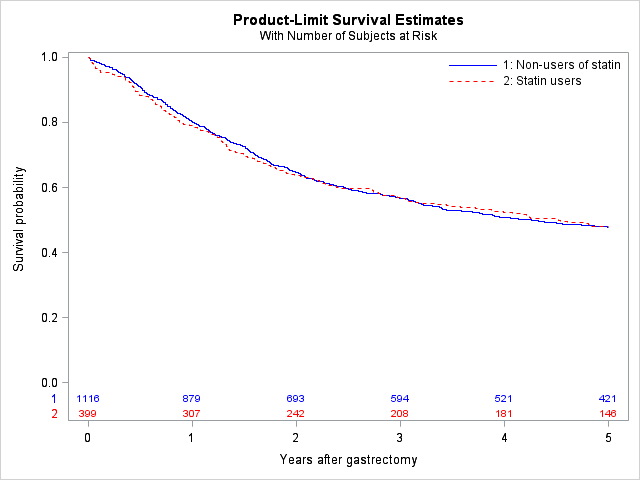


**Supplementary Figure 2. Cumulative incidence of 5-year all cause-specific mortality in patients with and without statins.**


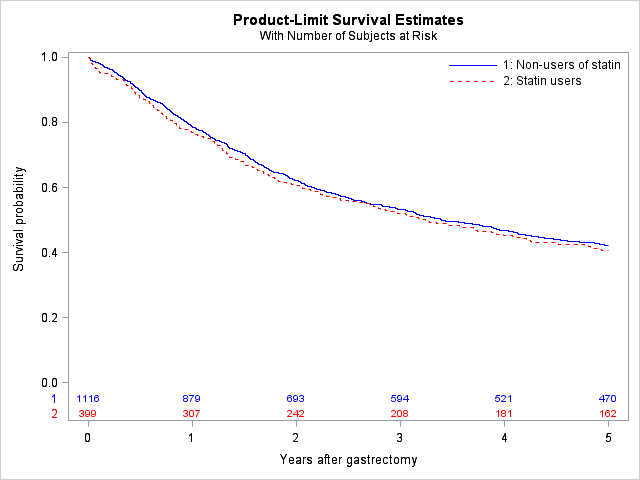

Supplement: Supplementary file 1 — Supplementary file1 (DOCX 51 KB) [file 10120_2024_1487_MOESM1_ESM.docx]
